# ASTER: A Package for Large-Scale Phylogenomic Reconstructions

**DOI:** 10.1093/molbev/msaf172

**Published:** 2025-07-16

**Authors:** Chao Zhang, Rasmus Nielsen, Siavash Mirarab

**Affiliations:** Globe Institute, University of Copenhagen, Øster Voldgade 5-7, Copenhagen 1350, Denmark; Integrative Biology Department, University of California Berkeley, 110 Sproul Hall, Berkeley, CA 94704, USA; Bioinformatics and Systems Biology, University of California San Diego, 9500 Gilman Drive, La Jolla, CA 92093, USA; Globe Institute, University of Copenhagen, Øster Voldgade 5-7, Copenhagen 1350, Denmark; Integrative Biology Department, University of California Berkeley, 110 Sproul Hall, Berkeley, CA 94704, USA; Electrical and Computer Engineering Department, University of California San Diego, 9500 Gilman Drive, La Jolla, CA 92093, USA

**Keywords:** phylogenomics, ASTRAL, species tree inference, quartet-based methods

## Abstract

Many algorithms are available for inferring species trees from various input types while accounting for gene tree discordance. Several quartet-based species tree inference methods, collectively known as the ASTRAL family, are based on similar ideas and are in wide use. Here, we integrate all ASTRAL-like methods into a single package called ASTER, comprising several tools, each designed for a different input type: (i) ASTRAL for single-copy gene tree topologies, (ii) weighted ASTRAL (wASTRAL) for single-copy gene tees with branch length and/or support, (iii) ASTRAL-Pro for multi-copy gene tree topologies, (iv) CASTER for multiple sequence alignments, including genome alignments, and (v) WASTER for short-reads and assembled genomes. These tools collectively enhance the scalability, accuracy, and versatility of species tree inference.

Inferring species trees from genome-wide data while accounting for gene tree discordance (Maddison 1997; Degnan and Rosenberg 2009) is enabled by a host of methods for various inputs (Blischak et al. 2023). Owing to theoretical advantages (Allman et al. 2011), many of these methods operate by defining a statistically consistent score per four species (a quartet) and finding the species tree with the maximum score summed over all the (n4) quartets. We have used this strategy to create the ASTRAL method (Mirarab et al. 2014) and a set of methods inspired by it (Zhang and Mirarab 2022a, 2022b; Zhang et al. 2018, 2020, 2025; Rabiee et al. 2019). What connects these methods is that they all optimize a score that is proved to be statistically consistent under the multi-species coalescent (MSC) model of incomplete lineage sorting (ILS) for a quartet and is then summed over all quartets; they optimize this sum without ever listing or enumerating all quartets and without subsampling quartets.

## ASTER

**Fig. 1.**
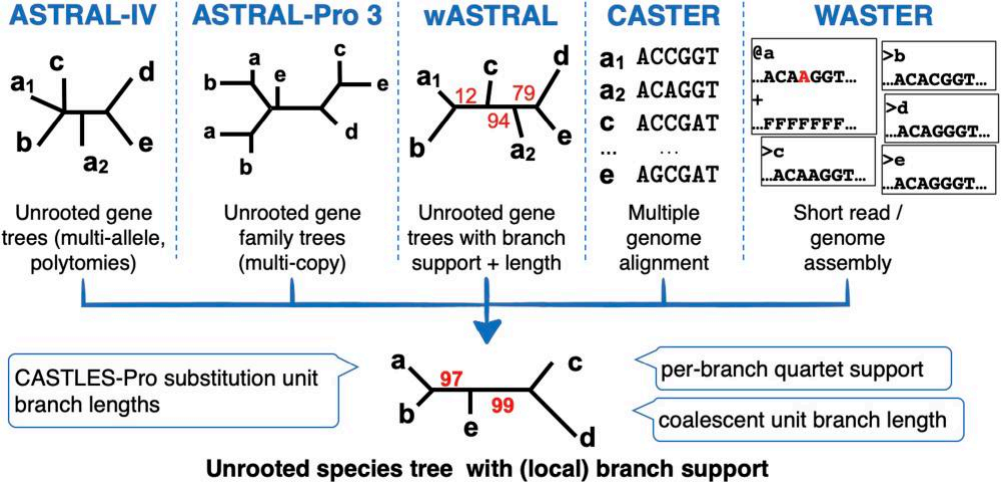
Overview of ASTER package. Each tool has a different input; all tools generate the species tree with support values. The output tree can be annotated by branch lengths in substitution units (for ASTRAL-IV and ASTRAL-Pro), coalescent unit lengths, and quartet support.

Here, we introduce the ASTER (Accurate Species Tree EstimatoR) suite of species tree inference tools, which consolidates our previously published ASTRAL-inspired tools. Unlike prior implementations, ASTER leverages the same underlying strategy to find the optimal species tree topology for all these methods, which differ in their input types and optimization criteria. The underlying search algorithm, called ASTER hereafter and first introduced by Zhang and Mirarab (2022b) for weighted ASTRAL, is designed to find the unrooted cladogram maximizing a score summed over all induced quartet trees without explicitly computing the score for each quartet. The ASTER algorithm builds upon but deviates from the original ASTRAL algorithm (Mirarab et al. 2014) in order to improve scalability with the number of genes and missing data. It is comprised of (i) the greedy inference of a set of *initial* trees using the sequential placement of species into a growing tree, (ii) nearest-neighbor interchange (NNI) moves to improve each initial tree, and (iii) dynamic programing akin to ASTRAL to find an optimal tree drawing its tripartitions from initial trees; in addition, a divide-and-conquer strategy is used to further improve the speed. ASTER currently comprises five tools that differ in their input and optimization criteria (Fig. 1).

### ASTRAL-IV

ASTRAL (Mirarab et al. 2014; Mirarab and Warnow 2015; Zhang et al. 2018) is a widely used tool for estimating an unrooted species tree from a set of unrooted single-copy gene trees (extended later by Rabiee et al. 2019 to handle multiple individuals per species). ASTRAL was designed for statistical consistency under the MSC model. ASTRAL-IV is a re-implementation of ASTRAL that adopts the ASTER algorithm, leading to following four advantages: (i) ASTRAL-IV scales linearly with the number of genes *k* compared to super-quadratically for ASTRAL-III ($O(k^{2.73})$ in the worst case and around quadratically in practice) and can thus handle a larger number of input trees. For example, ASTRAL-IV infers a phylogeny of 363 bird species from 63,430 gene trees of Stiller et al. (2024) in just 2 h using 32 CPU cores, as opposed to 31 h with 4 NVIDIA P100-16G GPUs and 28 CPU cores with the GPU-enabled ASTRAL-MP (Yin et al. 2019) implementation of ASTRAL-III. (ii) ASTRAL-IV handles missing data better. Zhang and Mirarab (2022b) compared the ASTER optimization strategy against that of ASTRAL-III and found that without missing data, ASTRAL-III is faster and finds better scores in difficult conditions, while ASTRAL-IV is faster and more effective in the presence of missing data, even at low levels. Due to better handling of missing data, ASTRAL-IV can also be used to compute a super-tree from a set of source trees (not necessarily gene trees, but also alternative incomplete species trees). (iii) ASTRAL-IV handles multi-individual data more efficiently. (iv) ASTRAL-IV integrates CASTLES-Pro introduced by Tabatabaee et al. (2025) and, as a result, computes both terminal and internal branch lengths in substitution-per-site units (ASTRAL-III can only compute coalescent unit lengths for internal nodes, which ASTRAL-IV can also output).

ASTRAL-IV, like all methods operating on gene tree topologies alone, can be sensitive to errors in gene tree estimation. A common strategy to mitigate these errors has been to contract gene tree branches with low support values (Mirarab 2023), a practice we also recommend for ASTRAL-IV to infer the topology (fully resolved gene trees are preferable for branch length estimation). We recommend obtaining gene tree support values using either bootstrapping or the much faster approximate Bayesian supports (aBayes), as implemented in IQ-TREE (Minh et al. 2020). While the optimal threshold of support to contract depends on the dataset, we have found low values (e.g. 10%) work well for bootstrap support (Zhang et al. 2018) while high values (e.g. 0.9) work well for aBayes support (Zhang and Mirarab 2022b; Mirarab 2023).

### Weighted ASTRAL (wASTRAL)

Handling low signals in gene trees using a threshold of support is not ideal. Zhang and Mirarab (2022b) introduced the alternative approach of weighting gene trees based on their branch lengths and/or support values (wASTRAL). By default, wASTRAL utilizes both branch lengths and branch supports, though it can use each individually. Simulations show that this weighting approach helps accuracy compared to contracting low-support branches (Zhang and Mirarab 2022b). We recommend wASTRAL over ASTRAL-IV because of its better handling of gene tree uncertainty. Just like ASTRAL-IV, it is sufficient to compute the local aBayes support for gene tree branches. Note, however, that wASTRAL does not compute substitution branch lengths; users can use the topology from wASTRAL as input to ASTRAL-IV to compute such branch lengths, with the caveat that this approach does not account for gene tree support.

### ASTRAL-Pro-3

ASTRAL-Pro (ASTRAL for PaRalogs and Orthologs) extends the capabilities of ASTRAL to accommodate multi-copy genes (Zhang et al. 2020). Its main conceptual difference from ASTRAL is that it tags internal nodes of gene trees as either duplication or (putatively) speciation and uses these tags to discount quartets resulting from paralogous copies; it also avoids double-counting multiple quartets that originated from the same speciation event, followed by subsequent duplications. Since ASTRAL-Pro2, the tool has been re-implemented using the ASTER algorithm, resulting in significantly reduced memory consumption and runtime (Zhang and Mirarab 2022a). ASTRAL-Pro3 further integrates CASTLES-Pro (Tabatabaee et al. 2025), allowing for the computation of both terminal and internal branch lengths in substitution-per-site units. Note that currently, no scalable algorithm has been devised to allow quartet weighting (similar to wASTRAL) for multi-copy genes, and thus, ASTRAL-Pro does not use gene tree branch support or branch length in inferring the topology. Moreover, for single-copy inputs with no support, while ASTRAL-Pro can be run if the input trees are fully resolved, we recommend that users continue to use ASTRAL-IV, which can handle polytomies. The calculation of local posterior probability handles missing data differently between ASTRAL-IV and ASTRAL-Pro3 (Zhang and Mirarab 2022a), and the ASTRAL-IV definition is more suitable for single-copy gene trees.

### CASTER

CASTER (Zhang et al. 2025) is a recently published coalescence-aware method that directly infers the species tree from a multiple sequence alignment. As a site-based method, CASTER bypasses the arbitrary division of genomes into supposedly recombination-free loci and avoids the costly inference of a separate gene tree for each such locus. Like ASTRAL, CASTER is a score summed over all quartets; however, the score for each quartet is a sum over all sites instead of a sum over all input gene trees. Its main innovation is defining a way to weight site patterns for each quartet to enable a statistically consistent estimator. Our simulations show that CASTER is 800 times less CPU-intensive than two-step methods (e.g. maximum likelihood for gene trees and weighted ASTRAL for the species tree) for 201 species and 10,000 loci, each 500 sites long, while achieving higher accuracy even under conditions of high ILS. Zhang et al. (2025) demonstrated that CASTER accurately infers the mammalian phylogeny using data from 241 whole genomes (exceeding 700 GB) on a single 64-core computer in 30 hours. Moreover, CASTER scores can be averaged across sliding windows to identify potential biological or artifactual signals (e.g. alignment errors). We recommend that users of ASTRAL who are working with whole genome alignments consider CASTER as an alternative (if gene trees are available) or replacement (when computing gene trees is infeasible).

CASTER comes in two flavors. The CASTER-site method uses site patterns, assumes the F84 model, and allows any form of mutation rate heterogeneity across the alignment. CASTER-pair models patterns across pairs of sites but assumes one of three 6-parameter “lumpable” sub-models of GTR (one is a generalization of TN93). While CASTER-pair allows a more general model and performs better in simulation, it has the downside of requiring us to pair sites, which may reduce the robustness to mutation rate heterogeneity. Also, the useful moving average feature is available only in the CASTER-site. Also, CASTER-site is faster. We suggest that when they can afford it, users should run both methods.

### WASTER

WASTER (Zhang and Nielsen 2025) is a coalescence-aware species tree inference tool designed to reconstruct shallow phylogenies directly from low-coverage short-reads. By bypassing the genome assembly and alignment steps, WASTER uses a k-mer-based approach to identify variable sites and defines the score based on patterns in these sites. Simulations show that WASTER achieves accuracy comparable to traditional alignment-based methods, even with coverage as low as 5X. In real datasets, WASTER successfully recovers phylogenetic trees that are originally inferred using alignment-based methods, from short-read data with only 1.5× coverage, substantially reducing the effort required for species tree estimation. WASTER can also generate guide trees for tree-based recursive alignment algorithms commonly used in large-scale analyses. However, due to its limitations, we do not recommend using WASTER trees as the primary source of evidence for deep evolutionary relationships. Future work will further attempt to achieve the same accuracy as alignment-based methods using alignment-free methods that build upon WASTER.

## Discussion and Future Work

ASTER provides a new C++ implementation of several tools currently in wide use. The consolidation makes the installation and use of these methods easier for users and makes the maintenance easier for developers. Integration of all these tools in one package also facilitates combining their features. For example, only ASTRAL-IV and ASTRAL-Pro 3 can produce substitution unit branch lengths currently; a user of wASTRAL that needs such lengths can still annotate branches of their wASTRAL tree using the ASTRAL-IV tool. ASTRAL-IV can also annotate branches with coalescent unit lengths and with quartet support.

The design of the ASTER software tool enables us to expand it in the future with new methods that optimize other quartet-based scores. A significant limitation of all the tools integrated so far is that none model gene flow and hybridization. In fact, the central theorem underpinning all of them, matching of the quartet species tree and the most frequent quartet gene tree, does not hold true for some gene flow scenarios (Solís-Lemus et al. 2016). It remains to be seen if this limitation can be resolved in the future by optimizing other scores. Another direction of future expansion is to incorporate other data types. For example, we have made available a new, unpublished algorithm called SISTER, which is currently in alpha release. SISTER is designed to take advantage of insertions and deletions rather than focusing on nucleotide or amino acid substitutions. Similar additions will be integrated into ASTER in the future as new algorithms are developed, welcoming contributions from the open-source community.

## Data Availability

The up-to-date ASTER package is available under AGPL at https://github.com/chaoszhang/ASTER and via Bioconda at https://bioconda.github.io/recipes/aster/README.html.
